# How might we build limbs *in vitro* informed by the modular aspects and tissue-dependency in limb development?

**DOI:** 10.3389/fcell.2023.1135784

**Published:** 2023-05-22

**Authors:** Rio Tsutsumi, Mototsugu Eiraku

**Affiliations:** ^1^ Institute for the Advanced Study of Human Biology (ASHBi), Kyoto University, Kyoto, Japan; ^2^ Laboratory of Developmental Systems, Institute for Life and Medical Sciences, Kyoto University, Kyoto, Japan

**Keywords:** limb organoid, stem cell engineering, limb development, pluripotent stem cells, morphogenesis

## Abstract

Building limb morphogenesis *in vitro* would substantially open up avenues for research and applications of appendage development. Recently, advances in stem cell engineering to differentiate desired cell types and produce multicellular structures *in vitro* have enabled the derivation of limb-like tissues from pluripotent stem cells. However, *in vitro* recapitulation of limb morphogenesis is yet to be achieved. To formulate a method of building limbs *in vitro*, it is critically important to understand developmental mechanisms, especially the modularity and the dependency of limb development on the external tissues, as those would help us to postulate what can be self-organized and what needs to be externally manipulated when reconstructing limb development *in vitro*. Although limbs are formed on the designated limb field on the flank of embryo in the normal developmental context, limbs can also be regenerated on the amputated stump in some animals and experimentally induced at ectopic locations, which highlights the modular aspects of limb morphogenesis. The forelimb-hindlimb identity and the dorsal-ventral, proximal-distal, and anterior-posterior axes are initially instructed by the body axis of the embryo, and maintained in the limb domain once established. In contrast, the aspects of dependency on the external tissues are especially underscored by the contribution of incoming tissues, such as muscles, blood vessels, and peripheral nerves, to developing limbs. Together, those developmental mechanisms explain how limb-like tissues could be derived from pluripotent stem cells. Prospectively, the higher complexity of limb morphologies is expected to be recapitulated by introducing the morphogen gradient and the incoming tissues in the culture environment. Those technological developments would dramatically enhance experimental accessibility and manipulability for elucidating the mechanisms of limb morphogenesis and interspecies differences. Furthermore, if human limb development can be modeled, drug development would be benefited by *in vitro* assessment of prenatal toxicity on congenital limb deficiencies. Ultimately, we might even create a future in which the lost appendage would be recovered by transplanting artificially grown human limbs.

## 1 Introduction

The current advances in stem cell biology have enabled the differentiation of the limb bud mesenchyme from pluripotent stem cells (PSCs) such as embryonic stem cells (ESCs) or induced pluripotent stem cells (iPSCs) (Chen et al., 2017; Mori et al., 2019; Yamada et al., 2021). Those findings have provided us with hopes of engineering limb morphogenesis *in vitro* and utilizing regenerative medicine to rescue damaged limbs. Engineering limb morphogenesis should require recapitulating the developmental environment of the limbs *in vitro*. Importantly, the modularity of limb development, namely, independence from the development of other organs, might potentially facilitate the self-organization of patterns and structures without external guidance in culture (Schlosser and Wagner, 2004; Sasai, 2013). Therefore, understanding the modular aspects and the dependency on the external tissues in limb development would help to postulate what can be self-organized and what needs to be manipulated *in vitro*, and to formulate the method for engineering limbs. In this article, we will review the current knowledge on the modularity of limb bud morphogenesis in embryonic development and regeneration as well as its dependence on the externalenvironment of the limb. Based on knowledge of *in vivo* limb morphogenesis, we will discuss the feasibility of *in vitro* engineering of the limb.

Limb development initiates as epithelial-mesenchymal transition of the lateral plate triggered by FGF-signaling from ectoderm (Gros and Tabin, 2014). The mesenchymal cells migrate towards the ectoderm at the lateral trunk region, and the outlining ectoderm then differentiates into a signaling center called the apical ectodermal ridge (AER). Signaling interactions between AER and the limb bud mesenchyme regulate massive cell proliferation and patterning of the limb bud.

Once the limb bud is established, the morphogenesis of the limb proceeds in a semi-independent manner, as shown by the fact that limb bud morphogenesis could happen in an unusual part of the body in experimental and regenerative situations. In 1995, Cohn et al. showed that when a bead soaked with FGF or WNT is implanted into the flank region between the forelimb and hindlimb, an ectopic limb bud is induced and the established bud then independently develops into a complete additional limb (Cohn et al., 1995; Kawakami et al., 2001). These studies suggest that once a limb bud is established, the limb morphogenesis requires little, if any, specificity of the tissues proximal to the limb. Thus, the ability of ectopic limb development is one of the striking examples showing the modularity of limb development.

Regeneration of the limbs in urodele amphibians and larval anurans is another example showing the modularity of limb formation, as it can be interpreted as ectopic and heterochronic limb development. When the limb is amputated in those animals, the stump is immediately covered by the wound epidermis and the wound epidermis then serves as the signaling center called apical ectodermal cap (AEC), which functionally corresponds to AER of the developing limb bud (Stoick-Cooper et al., 2007). Underneath the AEC, the cells in the remaining tissues of the limb, including dermis, cartilage, muscles, Schwann cells, and other connective tissues undergo dedifferentiation (Kragl et al., 2009), and the tissue stem cells such as muscle satellite cells are activated (Sandoval-Guzmán et al., 2014). The dedifferentiated cells and the activated tissue stem cells undergo extensive proliferation forming a blastema of mesenchyme, which essentially resembles the developmental limb bud mesenchyme.

Interestingly, Endo et al. (2004) discovered that, in regenerative urodele amphibians, after one peels off a piece of the skin on the lateral side of the limb, reroutes the nerve to the skin wound, and grafts a piece of skin from the side of the limb contralateral to the wound site, a blastema is formed ectopically on the lateral side of the limb. As a result of these manipulations, called the “accessory limb model,” the ectopic blastema continues to grow and forms an additional limb, demonstrating the minimum sufficient conditions to initiate limb regeneration. It was later found that when the same operation is conducted on the lateral trunk, ectopic formation of a blastema and limb morphogenesis can be induced even on the trunk (Hirata et al., 2013). These examples in highly regenerative animals suggested that the proper limb morphogenesis could happen not only in the designated limb field on the flank of the embryo, but also on the completely mature tissues of the adult limb and even the trunk if only the minimal conditions to trigger limb morphogenesis are satisfied.

Those instances highlight the autonomous and modular nature of limb morphogenesis. However, that does not mean limb morphogenesis could occur independently of the proximal tissues. Indeed, the *ex vivo* culture of chick limb bud fails to develop properly (Strangeways et al., 1926). What kinds of interactions between external tissues are important for limb development and how much independence does the developing limb have? To explore these questions, in the next section, we will review the molecular mechanisms of modularity, namely, how polarities and positional identities are established and maintained and their relationship with the basal tissues.

## 2 How polarities and positional identities are established?

The identity of limbs as forelimbs and hindlimbs is determined by the position along the rostrocaudal axis of the lateral plate where limb bud mesenchyme is derived (Isaac et al., 1998; Logan et al., 1998; Ohuchi et al., 1998). The positional identity of the lateral plate is governed by the antagonistic gradient of rostral retinoic acid (RA) and caudal FGF signaling (Ribes et al., 2009; Zhao and Duester, 2009). RA is required for the expression of *Tbx5* in the presumptive forelimb field of the lateral plate (Zhao et al., 2009). On the other hand, the hindlimb field in the caudal lateral plate is determined by GDF11 secreted from the paraxial mesoderm. GDF11 activates the expression of the hindlimb field and hindlimb bud-specific genes such as *Isl1*, *Pitx1*, and *Tbx4* in the lateral plate, and thus the initiation of *Gdf11* expression controls the position of the hindlimb along the body (McPherron et al., 1999; Jurberg et al., 2013; Matsubara et al., 2017). The anatomical features specific to the forelimb and hindlimb are specified by the expression of *Tbx5* in the forelimb and *Tbx4/Pitx1* in the hindlimb (Logan et al., 1998; Logan and Tabin, 1999; Rodriguez-Esteban et al., 1999; Takeuchi et al., 1999).

At the later stage of limb development, the same antagonistic gradient of RA and FGF controls the proximodistal identity of the limb bud, namely, stylopod, zeugopod, and autopod, from proximal to distal. This was first suggested in amphibian limb regeneration. When the limb is amputated, the lost limb element will be regenerated depending on the position of the amputation along the proximodistal axis. However, when a limb is treated with RA, the regenerated structure is proximalized relative to the position of amputation in a dose-dependent manner, resulting in a serial proximodistal duplication such that the stylopod is regenerated onto the remaining zeugopod (Niazi and Saxena, 1978; Maden, 1982; Maden, 1983). On the other hand, when blastema of a salamander formed on autopod is transplanted onto stylopod, zeugopod is regenerated in between (Iten and Bryant, 1975; Stocum, 1975; Maden, 1980; McCusker and Gardiner, 2013). This process is referred to as “intercalation”: when the adjacent cells have discontinuous positional information, the intermediate cells are regenerated to fill the gap in the positional identities (French et al., 1976; Agata et al., 2007). The mature autopod tissue is not capable of inducing intercalary regeneration between itself and the stylopod, but FGF treatment of the autopod endows the competency to induce the intercalary formation of zeugopod, suggesting that FGF might be the distal factor (Satoh et al., 2010).

It was later shown in chicken limb development that the transplanted limb bud mesenchyme after exposure to RA exhibits expansion of expression of the proximal marker, Meis1, and develops the proximalized structure. In contrast, exposure to FGF and Wnt, which is secreted from AER in normal development, expands the expression of the distal Hox gene *Hoxa13*, and distalizes the limb (Cooper et al., 2011; Roselló-Díez et al., 2011). Thus, even after the limb bud is initiated, proximal RA signaling from the trunk is supposedly required for the establishment of proper proximo-distal polarity, although there is controversy about this since in Rdh10 mutant mouse, which lacks RA synthesis, proximal-distal patterning of the hindlimb was not affected (Cunningham et al., 2013).

The key player in establishing the anteroposterior axis of the limb is SHH. The classical experiments using chick embryos strikingly showed that after transplantation of the tissue from the posterior margin of the limb bud, which is known as the zone of polarizing activity (ZPA), to the anterior margin of another limb bud, the mirror-duplicated pattern of three digits is induced along the anteroposterior axis (Saunders and Gasseling, 1968). It was later identified that SHH is the molecular entity of ZPA activity (Riddle et al., 1993). SHH, which is secreted from the posterior margin of the limb bud, specify anteroposterior identity of the digits in its early time window of the expression, and also promotes the expansion of the limb in its broader expression duration (Towers et al., 2008; 2011; Zhu et al., 2008; Zhu et al., 2022).

How is the posterior-biased expression of *Shh* established and how does the anteroposterior axis of limbs correspond to that of the body? The axial expression of *Hox9* paralogous group genes is restricted to the posterior portion of the forelimb-forming region and *Hox9* genes activate the expression of *Hand2* in the posterior forelimb, which then activates *Shh* in ZPA (Xu and Wellik, 2011). In contrast, the *Hox5* paralogous group genes repress the *Shh* expression in the anterior forelimb (Xu et al., 2013). Although these regulations by *Hox5* and *9* genes only apply to the forelimb, the expression domains of *Shh* in forelimb and hindlimb buds are regulated by the common downstream effectors such as the anterior repressor, *Gli3*, and the posterior activator, *Hand2* (Büscher et al., 1997; Galli et al., 2010). In hindlimb, the anterior expression of *Gli3* is regulated by *Sall4*, and the posterior expression of *Hand2* is regulated by *Isl1* (Itou et al., 2012; Akiyama et al., 2015). Interestingly, an additional limb induced in the trunk shows the inverted anteroposterior identity. This might be explained as occurring because the interlimb region posterior to the forelimb field, which would be the anterior portion of the additional limb, has stronger potency of polarizing activity (Tickle, 2015).

In amphibians, the interaction between anterior and posterior tissues at the stump is essential to initiate limb regeneration. If an anterior half of one limb is transplanted on an anterior half of another limb so that the stump has only either anterior identity, the limb fails to be regenerated (Bryant and Baca, 1978; Stocum, 1978). Conversely, if a left limb blastema is grafted onto a right stump so that the anterior tissues of the graft are adjacent to the posterior tissues of the stump, two supernumerary limbs are induced due to intercalation along the anteroposterior axis (Iten and Bryant, 1975). The accessory limb model, mentioned above, also requires a skin graft from a posterior limb onto an anteriorly created wound to initiate intercalation on the stump (Endo et al., 2004). The posterior skin can be substituted by RA or its downstream target, *Shh* (McCusker et al., 2014; Nacu et al., 2016; Iwata et al., 2020).

Limbs develop on the dorsoventral border of the trunk and AER is induced at the interface of dorsal and ventral ectoderm (Altabef et al., 1997). Dorsal ectoderm expresses *Wnt7a* (Parr and McMahon, 1995), whereas ventral ectoderm expresses *Bmp* (Pizette et al., 2001). Recombinant experiments performed by placing ectoderm in dorsoventrally reversed orientation with respect to the mesoderm showed that the signals from ectoderm specify the dorsoventral identity of the limb mesenchyme (MacCabe et al., 1974; Akita, 1996). Like their induction along the anteroposterior axis, supernumerary limbs can be induced by intercalation along the dorsoventral axis (Iten and Bryant, 1975).

How are positional identities interpreted into the anatomical complexity of limbs? Recent single-cell RNA-seq and lineage tracing experiments have shown that *Msx*-positive naïve limb progenitor cells differentiate into proximal or autopodial progenitors, while the naïve progenitors remain in the distal-most region beneath the AER. Three axes of positional identities affect the differentiation timing of proximal and autopodial progenitors in a complex manner seemingly underlying the mechanisms by which the positional identities are interpreted into the anatomical complexity of limb skeletons (Markman et al., 2023). A comprehensive understanding of this question requires an understanding of the differences in cellular behaviors in three-dimensional space in response to positional identities, as well as physical and mathematical approach to integrate collective cell behaviors into tissue-level morphogenesis. Limb organoids would be powerful model systems for the integral understanding of morphogenesis because simple aspects of three-dimensional morphogenesis can be extracted on a smaller scale in a more experimentally accessible environment.

**FIGURE 1:**
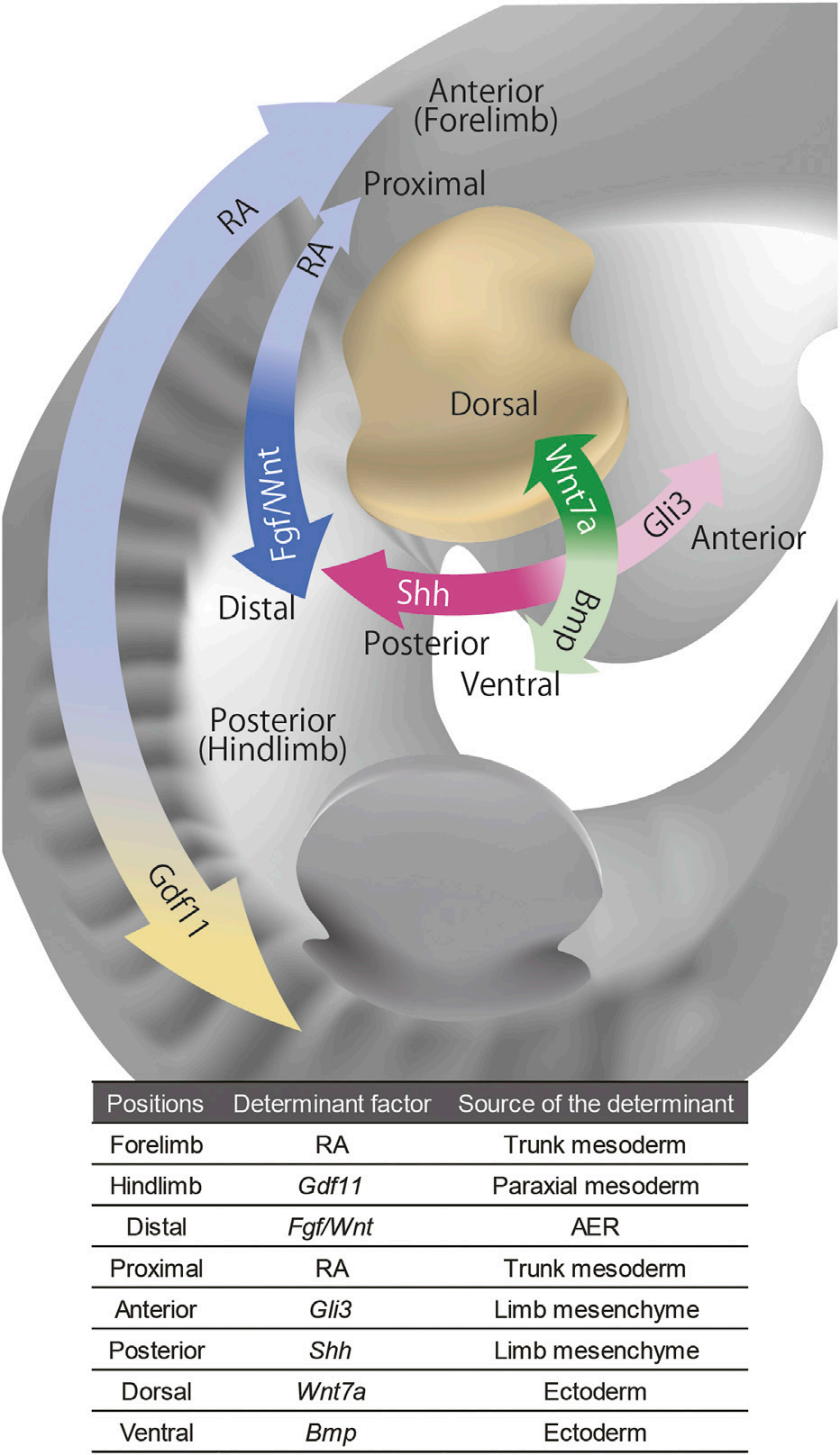
The signaling molecules that govern positional information of the limb bud. The distal-proximal axis of the limb bud is governed by FGF/WNT from AER and RA from trunk, the anterior-posterior axis is governed by *Shh* and *Gli3 from limb mesenchyme*, and the dorsal-ventral axis is governed by *Wnt7a* and *Bmp* from the ectoderm. The anterior-posterior axis of the whole body that governs the forelimb and hindlimb identities is regulated by RA and *Gdf11* in axial mesoderm.

To summarize this section, forelimb-hindlimb identity is determined by the positional identity of the lateral plate along the rostrocaudal axis. The proximo-distal axis is established through interactions between the trunk, or proximal limb, and distal AER. The anteroposterior axis of the limb is induced by the axial rostrocaudal identity. The dorsoventral axis is instructed by the dorsoventral identity of the ectoderm (Figure 1).Importantly, the fate map and transplantation experiments show that once the limb polarity is established, the positional identity can be maintained in a cell-autonomous manner without reference to the rest of the embryo. (reviewed in (Tickle, 2015)). However, the source of the limb tissues is not just the limb bud mesenchyme and the overlying ectoderm, but also incoming tissues from the trunk after the limb bud is initiated. In the next section, we will review how those incoming tissues acquire morphological information and how those tissues affect the morphogenesis of the other tissues.

## 3 The contribution of incoming tissues on limb morphogenesis

Muscle cells in the limb are derived from ventral–lateral somites and migrate into the nascent limb bud (Chevallier et al., 1977; Christ et al., 1977; Hayashi and Ozawa, 1991). The migration of muscle precursor cells is induced by Hgf expressed in limb mesenchyme (Bladt et al., 1995; Brand-Saberi et al., 1996; Scaal et al., 1999). Once they have migrated into the limb bud, the muscle precursors cluster into dorsal and ventral muscle masses (Hayashi and Ozawa, 1991). Subsequently, these muscle masses are subdivided into individual, anatomically distinct muscles by muscle connective tissues, which are derived from limb bud mesenchyme (Kardon, 1998; Kardon et al., 2003).

The specific patterns of individual muscles are governed by muscle connective tissues, and their surrounding blood vessels (Grim, 1991; Tozer et al., 2007; Mathew et al., 2011). Thus, muscle patterning defects occur due to mutation of the genes expressed in limb mesenchyme, such as *Tbx3*, *4*, and *5* (Hasson et al., 2010; Colasanto et al., 2016). Tendons are also derived from limb bud mesenchyme and are reciprocally dependent on muscles for proper patterning and maintenance (Christ et al., 1977; Kieny and Chevallier, 1979; Kardon, 1998; Schweitzer et al., 2001; Huang et al., 2015).

Although the initial patterning of bones and cartilage is not dependent on muscles, once the muscles are shaped into a functional unit together with muscle connective tissues and tendons, the mechanical forces applied to skeletons are responsible for proper bone morphogenesis, including longitudinal growth, circumferential growth, and formation of eminence (reviewed in (Felsenthal and Zelzer, 2017)). Strikingly, most of the limb joints fail to be matured and become fused (Pai, 1965; Rot-Nikcevic et al., 2006; Kahn et al., 2009; Nowlan et al., 2010).

Blood vessels are the incoming tissues that substantially affect the morphogenesis of the limb skeleton. Vascularization of the limb bud is initiated as angiogenesis from the dorsal aorta (Seichert and Rychter, 1972a; Seichert and Rychter, 1972b). Concomitantly, somite-derived angioblasts migrate into the limb bud and participate in vasculogenesis (Ambler et al., 2001). The importance of vasculature in limb skeletal morphogenesis is well demonstrated in avascular culture on chorioallantoic membrane (CAM). Chick limb bud graft could develop recognizable limb elements after being transplanted onto CAM, where diffusible nutrients, growth factors, and blood vessels are supplied from the host (Murray and Patrick, 1926). However, if the graft and CAM are separated by an intervening porous filter so that vascular invasion is prevented, the grafts will develop into very small and grossly distorted limbs (Searls, 1968).

The failure of proper cartilage morphogenesis in avascular culture does not seem to be simply due to the lack of oxygen and nutrient supply. Cartilage morphogenesis is associated with vascular remodeling since cartilage requires a hypoxic environment and hypoxic gene regulator, *Hif1a*, for its development and maintenance (Schipani et al., 2001; Amarilio et al., 2007; Provot et al., 2007; Bentovim et al., 2012). While blood vessels are distributed throughout the limb bud before the emergence of cartilage anlagen, localized vascular regression avascularizes the cartilage-forming area (Feinberg et al., 1986). Although this vascular remodeling is initiated by VEGF secreted from condensed mesenchyme (Eshkar-Oren et al., 2009), vasculatures reciprocally regulate skeletal morphogenesis (Yin and Pacifici, 2001). Furthermore, interdigital apoptosis requires reactive oxygen species produced in the highly vascularized area between digits (Eshkar-Oren et al., 2015).

Unlike cartilage, bone requires vascularization. Limb bone formation occurs via endochondral ossification, in which cartilage anlagen is replaced by bone minerals. At the center of the cartilage anlagen of long bones, the chondrocyte becomes hypertrophic chondrocyte and secretes VEGF to attract invasion of blood vessels (Gerber et al., 1999; Zelzer et al., 2002). Bone minerals are deposited by osteoblast precursors migrated from perichondrium/periosteum along with blood vessels (Maes et al., 2010), although recent evidences show trans-differentiation from hypertrophic chondrocytes is another source of osteoblasts (Yang et al., 2014a; Yang et al., 2014b; Zhou et al., 2014; Park et al., 2015). The vasculature is not only important for longitudinal bone growth, but it also serves as a guiding template for circumferential bone growth (Shoham et al., 2016).

Peripheral nerves are critically important for limb regeneration. It has long been known that if a limb of urodele amphibians is denervated, the amputated limb will fail to regenerate (Todd, 1823). Conversely, the existence of nerves at the wound site is one of the sufficient conditions for inducing ectopic limb regeneration in the accessory limb model (Endo et al., 2004). Rescue experiments in a denervated limb or accessory limb model without nerve rerouting showed that *nAG*, *Fgf,* B*mp*, and *Nrg1* are considered to be “nerve factors” which are secreted from peripheral nerves and induce blastema formation at a stump (Kumar et al., 2007; Makanae et al., 2014; Farkas et al., 2016). However, the nerve dependence seems to be specific to limb regeneration, and not to apply to limb development. When a chick limb bud graft is made such that the graft is not innervated from the host, musculoskeletal development of the grafts is grossly unaffected (Murray and Patrick, 1926; Hunt, 1932; Hamburger, 1939). Furthermore, even in limb regeneration, if a limb bud has never experienced innervation, the limb is competent for regeneration in the absence of nerves (Yntema, 1959). Thus, the requirement for peripheral nerves in limb formation might be contingent on the cellular context of limb progenitors.

**FIGURE 2:**
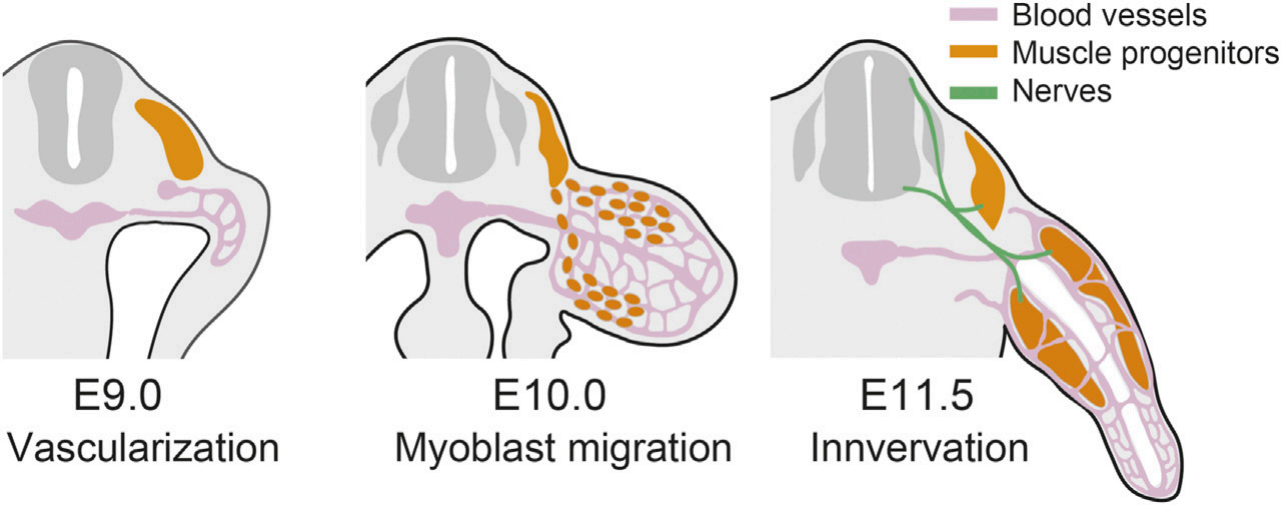
Contribution of the incoming tissues in limb development. In mouse forelimb, blood vessels invade the limb bud at E9.0 (Walls et al., 2008), muscle progenitors migrate into the limb at E10.0 (Houzelstein et al., 1999), and the limb bud is innervated at E11.5 (Hurren et al., 2015).

In this section, we argued that besides limb bud mesenchyme, tissues such as blood vessels, muscles, and peripheral nerves come into the limb bud and contribute to limb morphogenesis (Figure 2). Musculoskeletal morphologies are fine-tuned based on physical and biochemical interactions in multiple tissues, including muscles and peripheral nerves, which reinforce developmental robustness and malleability of limbs (reviewed in (Tsutsumi et al., 2017)). The invasion of blood vessels plays critical roles in initial skeletal patterning. Thus, among the incoming tissues, blood vessels are seemingly especially important for regulating the morphogenesis of limb mesenchyme.

In the context of engineering limb morphogenesis *in vitro*, is it necessary to direct the morphogenesis of those incoming tissues separately? Since limb morphogenesis is dependent on the interaction of multiple different tissues in the limb, it would be important to establish methods for co-culturing and integrating multiple cell lineages, including limb mesenchyme and the incoming tissues. However, the modular aspect of limb development and regeneration suggests that the incoming tissues as well as limb mesenchyme do not rely on continuous input of morphogenetic information from the external environment. Therefore, in the next section, we will discuss how to achieve the derivation of limb progenitor cells, introduce the positional identities of limb mesenchyme, and integrate them with the incoming tissues.

## 4 How might we build limbs *in vitro*?

**FIGURE 3:**
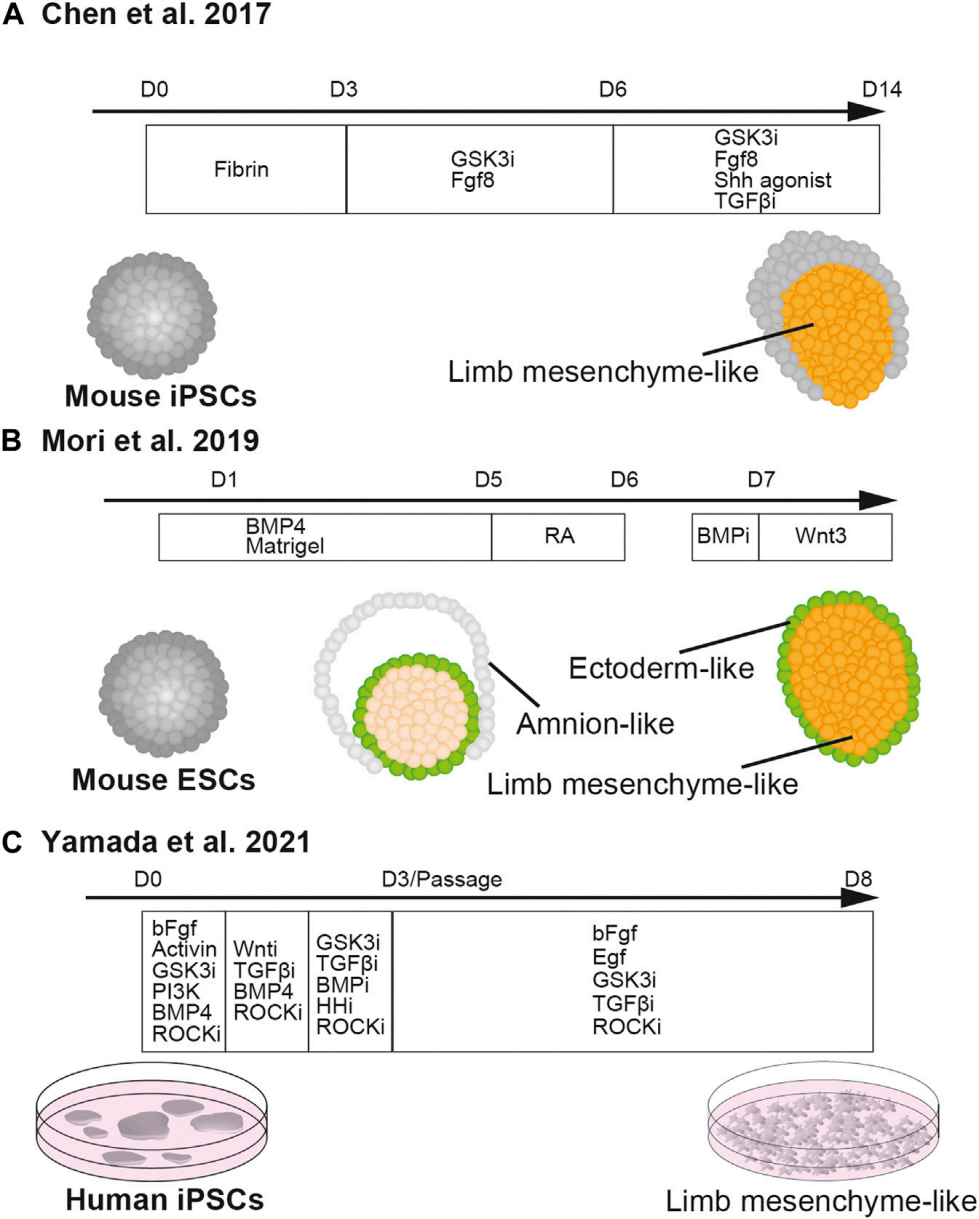
*In vitro* induction of limb mesenchyme from pluripotent stem cells.Chen et al., 2017 described the method of differentiating cell aggregates of mouse iPSCs into limb mesenchyme **(A)**. Mori et al., 2019 reported the differentiation of cell aggregates of mouse ESCs into limb mesenchyme wrapped by the ectoderm **(B)**. Yamada et al., 2021 established the method of differentiating human iPSCs into limb mesenchyme in two-dimensional culture **(C)**.

Recently, Chen et al. reported that three-dimensional culture of mouse iPSCs successfully induced the differentiation of cells with limb bud identity *in vitro* (Chen et al., 2017) (Figure 3A). Due to the high adhesiveness of PSCs, the cells in suspension culture are self-aggregated into embryoid bodies (Desbaillets et al., 2000). Chen et al. cultured the embryoid body with a WNT/β-catenin signaling agonist, CHIR99021, and FGF8 with a fibrin matrix for 6 days, and subsequently cultured it with SHH agonist Purmorphamine and a TGFβ type I receptor inhibitor, SB431542, together with CHIR99021 and FGF8. Both WNT signaling and FGF8 are present in the primitive streak and play important roles in the formation of mesoderm in the mouse embryo (Takada et al., 1994; Yoshikawa et al., 1997; Liu et al., 1999; Sun et al., 1999; Huelsken et al., 2000). Furthermore, treatment with the WNT/β-catenin signaling agonist induces mesoderm differentiation in the embryoid body (Berge et al., 2008; Brink et al., 2014). Thus, the first 6 days of treatment with the WNT/β-catenin signaling agonist and FGF8 seemed to drive mesodermal differentiation. While SHH has a critical role in the growth of limb bud mesenchyme, the TGFβ type I receptor inhibitor suppresses cartilage differentiation and expands undifferentiated limb bud mesenchyme (Parada et al., 2022).

Strikingly, the limb bud-like aggregates stimulated the regeneration of a mouse digit. Chen et al. transplanted the limb bud-like aggregates derived from iPSCs together with a cocktail of growth factors, FGF8, WNT3a, Thymosin β4, and BMP2 onto the amputated second phalange of an adult mouse digit. While the adult mouse is unable to regenerate the digit after amputation without any manipulation, the grafted digit showed the outgrowth of distal phalange tissues which were composed of both graft-derived and host-derived cells (Chen et al., 2017).

Later, it was reported that another three-dimensional culture method of mouse ESCs successfully recapitulated the differentiation of early limb bud-like structure *in vitro*, in which *Hand2*-positive mesenchyme is covered by *E-cadherin*-positive ectoderm (Mori et al., 2019) (Figure 3B). Essentially, in this study, mouse ESCs were differentiated using the serum-free floating culture of embryoid body-like aggregate with quick reaggregation (SFEBq) method with a high dose of BMP4. In SFEBq, ESCs are seeded in ultra-low adhesive 96-well plates to make embryoid bodies and differentiated in serum-free culture conditions (Eiraku et al., 2008). While without BMP, the embryoid body would spontaneously differentiate into epiblasts and subsequently neural ectoderm (Kamiya et al., 2011; Takata et al., 2016), a high dose of BMP might have contributed to fate decisions towards limb bud mesenchyme at multiple steps of lineage segregation. In the gastrulating mouse embryo, BMP4 secreted from extra-embryonic ectoderm regulates the formation of primitive streak from the epiblasts (Mishina et al., 1995; Winnier et al., 1995; Coucouvanis and Martin, 1999; Beppu et al., 2000). Thus, the first fate decision directed by BMP was to drive differentiation of ESCs towards mesoendoderm, instead of ectoderm (Johansson and Wiles, 1995; Finley et al., 1999; Ying et al., 2003; Ng et al., 2005).

After the primitive streak is established, mesodermal cells from the posterior part of the primitive streak migrate laterally more robustly than those from the anterior part, and thereby the anteroposterior identities of the primitive streak are converted into the mediolateral axis of mesoderm at the later embryonic stage. At this stage, BMP4 that is secreted from the lateral plate and NOGGIN, a BMP antagonist secreted from the notochord, constitute a BMP gradient along the mediolateral axis, specifying the subtypes of mesoderm (Pourquié et al., 1996; Tonegawa et al., 1997; Tonegawa and Takahashi, 1998). Thus, in the culture in Mori et al. (2019) the second fate decision directed by the high dose of BMP was to instruct differentiation of lateral plate mesoderm.

Although it is not very clear how the aggregate with lateral plate identity is differentiated into limb bud mesenchyme in the culture of Mori et al., 2019 the overlying ectoderm might induce epithelial-mesenchymal transition and differentiation of limb bud mesenchyme, as happens in the embryo (Gros and Tabin, 2014). As discussed above, the dorsoventral identity of the limb bud is specified by dorsal *Wnt7a* and ventral *Bmp*. Hence, the third fate decision by BMP was to grant ventral identity to the limb bud-like aggregates. Indeed, local inhibition of BMP signaling created a portion with dorsal identity in the aggregate and induced the formation of AER-like structure at the dorsoventral border.

Yamada et al. (2021) reported the derivation of limb bud-like cells from human iPSCs (Figure 3C). In their study, iPSCs were differentiated in a step-wise manner from the mid-primitive streak, lateral plate, and limb bud progenitors by daily changes of medium with composition modified from that described in Loh et al. (2016) so as to maximize the expressions of PRRX1 and other limb bud markers. The differentiated limb bud-like cells could be expanded by activation of EGF, FGF, and canonical WNT signaling, as well as suppression of TGF-β signaling. The limb bud-like cells, which have the potential to produce cartilage, have proven to be useful for drug screening of therapeutic candidates for treating type II collagenopathy (Yamada et al., 2021).

Chen et al. (2017); Mori et al. (2019), and Yamada et al. (2021) showed that the PSC-derived limb bud-like cells are capable of forming skeletal tissues after being transplanted in mouse or rat (Table. 1). However, their ability to undergo cartilage morphogenesis in culture has not been extensively explored. It should be noted that in the study of Mori et al., the anteroposterior and proximo-distal identities in the limb bud-like cells within the aggregate were disorganized. Though the positional identities of the cells in the aggregate were not addressed in Chen et al., given that among the four growth factors in the cocktail, FGF and WNT specify the distal identity of the limb bud, the interaction between the distally specified limb bud-like aggregate and the host tissues might have established the proximo-distal axis, which might have then enabled the organized outgrowth of the skeletal tissues. Furthermore, it can be speculated that blood vessels might have invaded the graft and stimulated the growth and morphogenesis of the regenerating distal digit.

**TABLE 1:** *In vitro* inductions of limb mesenchyme and their potency after transplantation experiments.

Paper	Source	Outcome	Transplantation experiment
Chen et al., 2017	Embryoid body from mouse iPSCs	Limb mesenchyme-like cells	Induced phalange regeneration after transplantation onto the P2 amputation in adult mice
Mori et al., 2019	Embryoid body from mouse ESCs	Limb mesenchyme-like cells wrapped by ectoderm	Contribution to multiple connective tissues after cellular engraftment
			Cartilage differentiation and endochondral ossification In the renal capsule
Yamada et al., 2021	Human iPSCs	Expandable limb mesenchyme-like cells	Formation of hyaline cartilage like-structure after subcutaneous transplantation to mouse and articular cartilage transplantation to rat

Is it possible to recapitulate more complex limb morphogenesis with cultures from PSCs? To achieve this goal, it seems essential to induce proper organization of the proximo-distal, dorso-ventral, and anteroposterior axis and to induce vascularization into the limb bud organoid. In fact, these are two of the current major challenges in the field of organoids in general: to provide morphogen gradients that are not self-organized in the aggregate and to induce vascularization.

To establish morphogen gradient in the culture environment of organoids, methods are selected based on the scale and duration of the gradient to be maintained. One commonly used method relies on diffusion from two reservoir chambers. Organoids are embedded in hydrogels and placed between the two chambers (Attayek et al., 2016; Tabata and Lutolf, 2017; Koh and Hagiwara, 2023). Using this method, a chemical gradient spanning 1.5-6 folds in a 1 mm scale can be achieved. When the media in two chambers are kept replenished or the chambers have a large enough volume, the gradient can be maintained for 5–10 days. An orthogonal gradient to create two axes (Such as dorsal-ventral and anterior-posterior) can be established by four microfluidic channels, where two parallel flows are placed perpendicular to each other (Demers et al., 2016).

When a smaller scale of the gradient is required, local administration of signaling molecules can be utilized. As mentioned above, Mori et al. (2019) injected a BMP inhibitor onto a limb bud-like aggregate through a glass capillary and successfully induced a dorsalized hemisphere within the aggregate. In this case, since the aggregate was globally ventralized by BMP signaling prior to the injection, local inhibition of BMP signaling could sufficiently establish a morphogen gradient. Embedding morphogen-secreting cells can generate a gradient with 500 μm scale (Cederquist et al., 2019). By using light-inducible expression of morphogen, more precise special control might be achieved (De Santis et al., 2021).

When a larger scale of the gradient is desired, a 4 mm scale of the gradient can be achieved by embedding organoids on the confluent of two parallel streams (Park et al., 2009). An even larger scale (−20 mm) of the gradient can be achieved by the perpendicular sequential diffusive mixing of two inlet media (Rifes et al., 2020)

Alternatively, the organoid assembly approach might be utilized. In this approach, two organoids with two different positional information are differentiated separately and combined into one “assembloid” (Bagley et al., 2017; Birey et al., 2017; Koike et al., 2019). This approach requires to the identification of the timing at which the positional identities of the two organoids are fixed while two organoids are still capable of fusing, but it is possible that this approach might recapitulate the situation of intercalary and supernumerary regeneration and might powerfully promote limb morphogenesis.

To vascularize organoids, one of the approaches is co-culturing with endothelial progenitor cells. For example, in a brain organoid, human iPS-derived endothelial cells formed a vascular network and improved the survivability and maturation of the organoid (Pham et al., 2018; Cakir et al., 2019). Similarly, vascularized organoids were also generated by co-culturing human PSC-derived cells and human umbilical vein endothelial cells (HUVECs), which are primary endothelial cells with high potential for blood vessel formation *in vitro* (Takebe et al., 2013; Isshiki et al., 2020; Shi et al., 2020). However, it is still a challenge to achieve functional perfusion of the vascularized organoids *in vitro*. This goal will likely be achieved by identifying culture environments which allow integration of a microfluidic device with a vascular bed and vascularized organoids (Zhang et al., 2021).

## 5 Conclusion

It is becoming technically feasible to introduce appropriate morphogen gradients exogenously and to introduce blood vessels and other tissues to limb bud organoids. If engineering a more complex limb bud could be achieved, it would substantially open up avenues for research and applications of appendage development. If we can create *in vitro* models of three-dimensional limb development, manipulability to elucidate the mechanisms of morphogenesis will be dramatically enhanced. Furthermore, since iPSCs have been established in many non-traditional model organisms, molecular and genetic mechanisms of mammalian limb diversities, including specific features of human limbs, are expected to be studied using organoids from PS cells. For instance, the extensive growth of the proximal hindlimb is one of the remarkable differences between the bodies of modern humans and any other extant animals. It has enabled our upright bipedalism and presumably allowed us to have a heavy brain and has released our hands from their roles for walking. Thus, it would be exciting to have an opportunity to unravel the genetic and developmental mechanisms of the evolution of our limb proportions. Finally, models of human limb development would bring about medical benefits, such as drug screening to assess prenatal toxicity causing congenital limb defects and ultimately rescuing lost appendages by transplanting in vitro-derived human limb bud tissues.
